# Facile Synthesis of Sprayed CNTs Layer-Embedded Stretchable Sensors with Controllable Sensitivity

**DOI:** 10.3390/polym13020311

**Published:** 2021-01-19

**Authors:** Hammad R. Khalid, Iqra Choudhry, Daeik Jang, Nadir Abbas, M. Salman Haider, H. K. Lee

**Affiliations:** 1 Civil and Environmental Engineering Department, King Fahd University of Petroleum and Minerals, Dhahran 31261, Saudi Arabia; 2 Department of Civil and Environmental Engineering, KAIST, 291 Daehak-ro, Yuseong-gu, Daejoen 34141, Korea; choudhry.iqra4@gmail.com (I.C.); svs2002@kaist.ac.kr (D.J.); haengki@kaist.ac.kr (H.K.L.); 3 Department of Chemical Engineering, University of Hai’l, Hai’l 81441, Saudi Arabia; n.abbas@uoh.edu.sa; 4 Department of Chemical Engineering, University of Gujrat, HH Campus, Gujrat 50700, Pakistan; salman.haider@uog.edu.pk or

**Keywords:** stretchable senor, flexible sensor, wearable sensor, functional composite, spray deposition, carbon nanotubes, piezoresistive material, polymeric composite

## Abstract

Flexible electronic devices have gained significant interest due to their different potential applications. Herein, we report highly flexible, stretchable, and sensitive sensors made of sprayed CNT layer, sandwiched between two polymer layers. A facile fabrication process was employed in which the CNT solution was directly sprayed onto a patterned bottom polymer layer, above which a second polymer layer was casted to get a sandwiched composite structure. Varying amounts of CNT solution (i.e., 10, 25, 40, 70, and 100 mL) were sprayed to get conductive CNT layers of different thicknesses/densities. The physical characteristics of the conductive CNT layers were studied through SEM and optical images. The starting electrical resistance values (without strain) as well as the changes in electrical resistance against human body motions were monitored. The synthesized samples exhibited good response against finger and wrist bending. The conductivity of the samples increased with increase of CNT solution volume while the sensitivity followed the inverse relation, suggesting that the sensors with controlled sensitivity could be fabricated for targeted strain ranges using the proposed method.

## 1. Introduction

Wearable electronic devices are getting increasing attention due to their several applications [1,2,3]. For such devices, sensors should possess high stretchability and sensitivity. In this regard, conductive fillers are normally incorporated into soft polymers to attain desired functionality [4,5,6]. Among others, CNTs are regarded as favorable conductive fillers possessing excellent sensing capabilities [7]. Nevertheless, dispersion of CNTs in polymers using conventional solution dispersion or a melting process is still a cumbersome task.

Recently, efforts were made to deposit conductive fillers onto polymer substrate using hot press [8], inkjet printing [9], or spray-deposition [10,11]. By doing so, a complete conductive layer/film could be obtained that generates signals upon stretching/releasing of the polymer. Mishra et al. [8] and Amjadi et al. [10] deposited a CNT film onto donor substrate which was then transferred to a recipient substrate. The samples showed good sensing response, however, it is difficult to ensure the integrity of the film as well as its complete transfer to the recipient substrate during the film transfer. Moreover, limited geometries could be created in the film transfer technique [9]. Lipomi et al. [11] spray-coated CNTs onto two PDMS layers and then joined them together using Ecoflex^TM^ silicone polymer, which served as a dielectric layer.

In this work, we report a facile fabrication process in which CNT solution was directly sprayed onto patterned polymer substrate, using a simple spray gun. Since quantity of CNTs is of vital importance in terms of electrical conductivity and sensitivity, varying amounts of CNT solution were sprayed to obtain conductive CNT layers with different thicknesses/densities. The deposited CNT layers were characterized by electrical resistance measurements and SEM/optical image analyses. Following this, the sensors were tested for detecting different human gestures.

## 2. Materials and Methods

The silicone polymer (Ecoflex^TM^ 00-30), multi-walled CNTs, and isopropyl alcohol (IPA) were procured from Smooth-on Inc. (Macungie, USA), Hyosung Inc. (Seoul, South Korea), and Samchun Chemicals (Seoul, South Korea), respectively. The tensile strength, specific gravity, and elongation at the break of polymer were 200 psi, 1.07, and 900%, respectively. The length, diameter, and specific gravity of CNTs were 10 μm, 12–40 nm, and 1.32, respectively.

**Figure 1:**
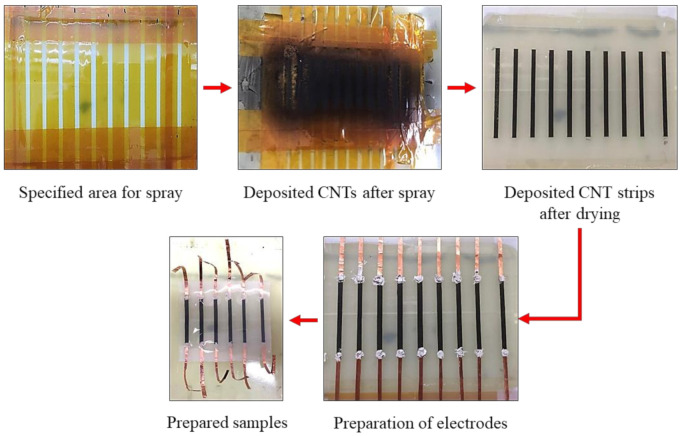
Fabrication process of sprayed CNT layer-embedded sensors.

The fabrication steps are shown in Figure 1. A glass plate, lined with scotch tape, was used as substrate to cast the polymer, while 1 mm thick glass slides were used to control the layer thickness. The polymer with same amounts of part A and B was poured in the designated area (120 × 70 mm^2^ to get ten samples, each sized 10 × 70 mm^2^) and was kept at 70 °C for 2 h. In parallel, the CNT solution was prepared by adding 0.05 g CNTs in 100 g IPA. The solution was sonicated for 1 h, followed by 1 h of stirring at 300 rpm. After 2 h curing of the bottom polymer layer, the spray areas were specified (3 × 50 mm^2^ for each CNT strip) by applying PI tape. An air sprayer, with an attached pressure gauge to control the spraying pressure (1 bar), was used. The glass plate was kept on a hot plate at 110 °C for rapid evaporation of IPA, which was essential for getting a uniform layer of CNTs. Five types of samples (S10, S25, S40, S70, and S100) were prepared by using 10, 25, 40, 70, or 100 mL of CNT solution, respectively. After spraying, the samples were kept at 80 °C for 30 min to ensure complete evaporation of IPA. Then, the electrodes were made using copper tape, followed by deposition of the top polymer layer to get a sandwiched structure. Lastly, individual samples were obtained by cutting with a paper cutter.

Multiple image analysis techniques (SEM and optical images using Digibird microscope OPT-500, Seoul, South Korea) were employed to visually examine the CNT layers. The electrical resistance values (non-strained) of three replicas of each sample type were recorded through a digital multimeter (DMM 34410A made by Agilent Technologies Korea Ltd., Seoul, South Korea) to investigate the connectivity of CNTs in the deposited layers. Following this, the efficiency of samples for detecting several human joint movements was explored to demonstrate the potential of as-synthesized samples to be used in wearable devices.

## 3. Results

**Figure 2:**
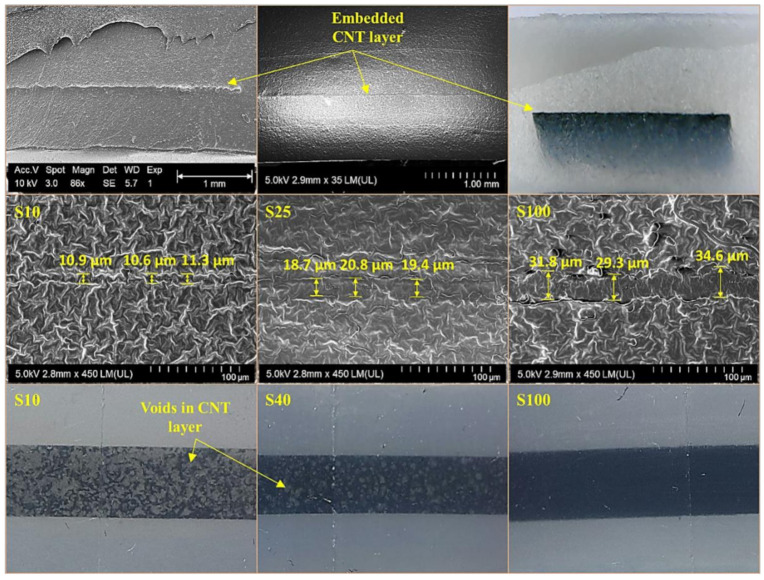
Image analysis of CNT layer-embedded sensors.

The SEM micrographs and optical images are shown in Figure 2. The CNT layer-embedded between two polymer layers can be clearly seen in these images. It was noted that as the sprayed volume of the CNT solution increased, the thickness (shown in SEM micrographs) and density (which can be visualized in optical images) of CNT layers also increased. The approximate CNT layer thickness was about 11 μm in sample S10, which increased to about 19 and 31 μm in samples S25 and S100, respectively. Optical images revealed that many voids were present in sample S10. The size and number of voids decreased in sample S40, while no visible voids were identified in sample S100. This could be attributed to the total amount of CNT solution used for each sample type. Seemingly, 10 mL of the CNT solution used for sample S10 was not enough to fully cover the spraying area which resulted in many voids. An increase in CNT solution amount decreased the voids in the CNT layers for the subsequent samples (S25 to S100). This also confirmed the good deposition of CNTs with increase in spraying volume, resulting in a uniform dense conductive layer.

The resistance values of the non-strained samples were recorded. Sample S10 showed a highly unstable resistance of about 21 MΩ. Moreover, it often went out of the range of the multimeter showing a complete loss of electrical path, resulting from the poor connectivity of CNTs. Hence, sample S10 was excluded from further analysis. As the spraying volume increased to 25 mL, a sudden decrease in resistance was observed. Samples S25, S40, S70, and S100 showed about 704, 41, 12, and 4 kΩ resistance values, respectively. This suggests that the CNTs were well-connected in the layer, ensuring a stable electrical path. These findings are compatible with the image analysis results. The thickness of the conductive layers and their voids can be associated with the observed resistance values. Based on these observations, it could be said that the connectivity of CNTs in the layer could be controlled by using a specific amount of spraying solution in this facile fabrication process, to achieve the targeted sensitivity and stability of the sensor.

**Figure 3:**
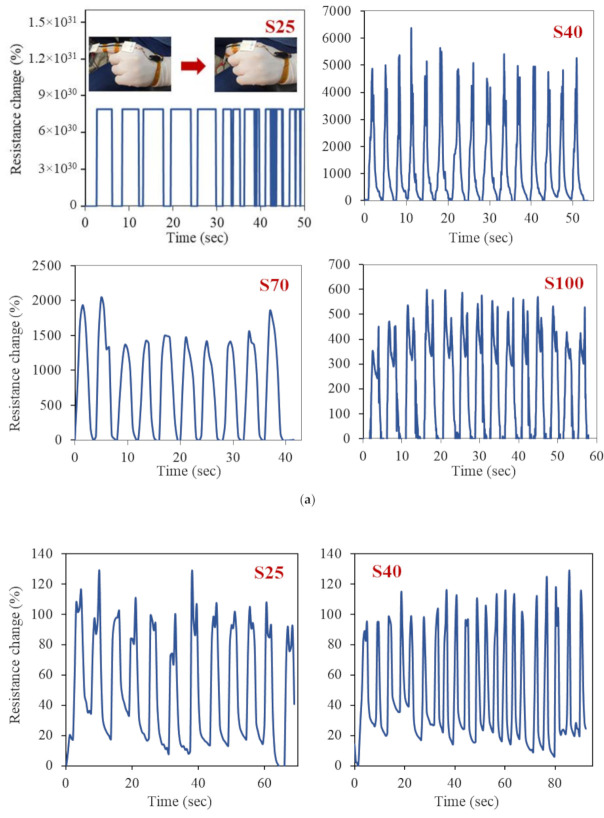

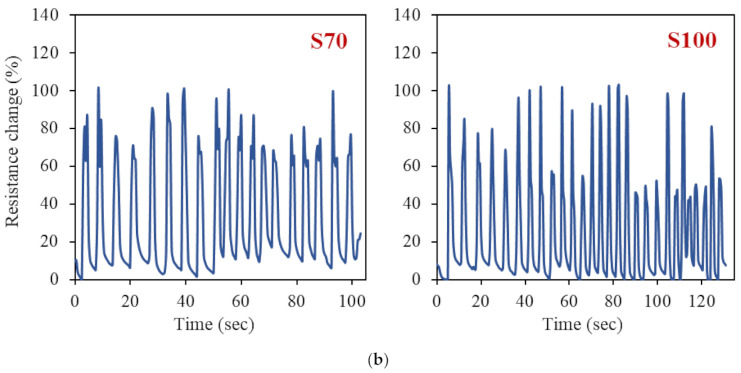
Electrical resistance change of sensors against finger (**a**) and wrist (**b**) bending.

Lastly, the samples were tested for detecting human joint movements. Specifically, finger and wrist bending were monitored by attaching the samples on index finger and wrist, respectively. The response of the samples in form of changes in resistance against finger and wrist bending are shown in Figure 3. It can be clearly seen that the samples exhibited good response against finger and wrist bending. Sharp increases in resistance were observed upon bending, which were immediately recovered upon straightening. It was further noted that change in resistance was related to the sprayed volume which in turn corresponded to the density of the CNT layer. Upon finger bending, the resistance of samples S25 exceeded the range of the multimeter, exhibiting a complete loss of electrical connection (Figure 3a). The average resistance changes of samples S40, S70, and S100 against finger bending were about 5000%, 1500%, and 500%, respectively. These resistance change values were much higher than the values reported in the literature [10,12]. This was achieved because of the low stiffness of silicone polymer and free movement of CNTs along with the stretching of the polymer, followed by their re-connectivity upon relaxation. Moreover, the sensitivity of the samples followed the order of S25 > S40 > S70 > S100. These findings suggest that the sensors efficiency greatly depended on the amount of sprayed volume, which controlled the electrical pathways through varying density and thickness of the CNT layer. Hence, the samples with targeted sensitivity could be fabricated for the specific strain ranges.

The sensors also showed a good response against wrist bending. Since the bend angle in wrist bending (~60°) was less than the finger bending (>90°), the change in resistance was normally lower than the change against the finger bending [10]. Similar behavior was observed in this study, with resistance change in the range of about 50–130% (Figure 3b). Although a slight decrease in resistance change was observed from samples S25 to S100, the change in the sensitivity of the samples was not that prominent as it was against finger bending. Samples 25 and S40 exhibited resistance change in the range of about 100–130%, while this value decreased to about 70–100% and 50–100% for samples S70 and S100, respectively. This could also be attributed to the less bend angle in wrist bending, resulting in less loss of electrical connections compared to more losses in finger bending. Finally, it was reported in the literature that the performance of such sensors degrades over time which is crucial for their long-term use [13], hence, should be considered in future studies.

## 4. Conclusions

In summary, the efficiency of the facile fabrication process was demonstrated. The conductive CNT layers were directly deposited onto the polymer. Using the proposed method, increasing amounts of CNTs could be easily spray-deposited, while avoiding issues like the non-uniform dispersion of CNTs in polymers, stiffening of the polymer itself, etc. The synthesized samples showed good sensing capabilities against different human motions. Furthermore, sensitivity of the samples was found to be dependent on the amount of CNT solution used. Hence, a specific amount of CNT solution could be used to achieve the desired sensitivity to target specific human motions.

## Data Availability

The data presented in this study are available on request from the corresponding author.
